# Exploring the prognostic impact of absolute lymphocyte count in patients treated with immune-checkpoint inhibitors

**DOI:** 10.1038/s44276-024-00058-6

**Published:** 2024-04-18

**Authors:** M. R. Conroy, H. O’Sullivan, D. C. Collins, R. M. Bambury, D. Power, S. Grossman, S. O’Reilly

**Affiliations:** 1 Department of Medical Oncology, Cork University Hospital, Cork, Ireland; 2 Cancer research @UCC, Western Gateway Building, Western Road, Cork, Ireland; 3 Mercy University Hospital, Grenville Pl, Centre, Cork, Ireland; 4 Johns Hopkins University, Baltimore, MD USA

## Abstract

**Background:**

The role of immune checkpoint inhibitors (ICI) expands but affordable and reproducible prognostic biomarkers are needed. We investigated the association between baseline and 3-month absolute lymphocyte count (ALC) and survival for patients on ICI.

**Methods:**

A retrospective study investigated patients who received ICI July 2014—August 2019. Survival probabilities were calculated for lymphocyte subsets. Univariate and multivariate analyses were performed to investigate risk factors for lymphopenia.

**Results:**

Among 179 patients, median age was 62 and 41% were female. The most common diagnoses were melanoma (41%) and lung cancer (40%). Median PFS was 6.5 months. 27% had baseline lymphopenia (ALC < 1 × 10^9^cells/L) and no significant difference in PFS or OS to those with normal ALC. However, 31% had lymphopenia at 3 months and significantly shorter OS than those without (9.8 vs 18.3 months, *p* < 0.001). Those with baseline lymphopenia who recovered counts at 3 months had no difference in PFS (median NR vs 13.0 months, *p* = 0.48) or OS (22 vs 18.3 months, *p* = 0.548) to those never lymphopenic. The strongest risk factor for lymphopenia on multivariable analysis was previous radiation therapy (RT).

**Conclusions:**

3-month lymphopenia is a negative prognostic marker in cancer patients on ICI. Previous RT is significantly associated with lymphopenia.

## Background

The use of immune checkpoint inhibitors (ICI) continues to expand, with a wider range of indications [1] but still at significant cost [2]. There is a persisting need for simple, affordable and accessible predictors of response. While the use of ICI has transformed outcomes for many cancers [3, 4], the majority of patients still do not experience a tumour response [5].

Existing biomarkers of response, including PD-L1 status, tumour mutational burden and mismatch repair status are inconsistently predictive of response and not practical in all cases [6–9].

In parallel, interest has grown in the relevance of lymphopenia in cancer. It has been known for decades that a significant proportion of cancer patients with a history of systemic anti-cancer therapy (SACT) or radiation treatment (RT) will develop severe lymphopenia [10, 11], which is independently associated with shorter survival in a range of cancers [12–16]. Other work has explored related haematologic indices, such as the neutrophil-to-lymphocyte ratio (NLR) and platelet-to-lymphocyte ratio (PLR). An elevated NLR at baseline has consistently been associated with poorer outcomes among cancer patients in general [16, 17], and among those treated with immunotherapy in particular [18–20]. A higher PLR has also been associated with inferior survival, most commonly among lung cancer patients on ICI [19, 21, 22].

We explored the relationship between absolute lymphocyte count (ALC) and survival for patients on ICI. We analysed both ALC at specified timepoints (baseline and three months) and change in ALC as a dynamic biomarker over that period. In addition, we investigated risk factors for lymphopenia in patients commencing ICI.

## Methods

A retrospective review of patients in Cork University Hospital and Mercy University Hospital was performed, identifying any patients treated with at least two doses of ICI for an approved indication over a 5-year period between July 2014 and August 2019. Solid tumour types included non-small cell lung cancer, melanoma, renal cell carcinoma, urothelial cancer, Merkel cell carcinoma, cervical cancer and mismatch repair-deficient (dMMR) colorectal and gastric cancer. We excluded patients who had received chemoimmunotherapy combinations or who had received ICI for haematologic malignancy. We gathered baseline demographic and pathological characteristics, bloodwork at treatment initiation and 3 months, treatment history (including RT), survival data and recorded treatment-related toxicities. PFS was measured from date of commencement of ICI to date of tumour progression, death or last follow-up. OS was measured from date of commencement of ICI to death or last follow-up. The interval of imaging studies was at the discretion of the individual oncologist but for most patients was approximately every 3 months.

Low ALC was graded according to the Common Terminology Criteria for Adverse Events (CTCAE) version 5. Grades one and two lymphopenia describe counts between the lower limit of normal and 0.5 × 10^9^cells/L, with grades three and four describing counts below 0.5 × 10^9^cells/L.

Survival probabilities and median survival with 95% confidence intervals (CI) were estimated according to the Kaplan–Meier method and compared using log-rank tests. Univariate analyses were conducted with Fisher’s exact test. Multivariable analyses were carried out with binary logistic regression. A time-varying Cox proportional hazards regression model was used to estimate the impact of the time-dependent changes in the ALC on survival. *P* values of less than 0.05 were considered statistically significant. All statistical analyses were carried out using SPSS (version 26; IBM Corp., Armonk, NY). Ethical approval for the study was obtained from the Clinical Research Ethics Committee of the Cork Teaching Hospitals.

## Results

Between July 2014 and August 2019, 179 patients who met the inclusion criteria were identified. Their baseline characteristics are summarised in Table 1. Median age at diagnosis was 62 years (range 19 to 83). Seventy-four (41%) were female. The most common cancers were melanoma (74, 41%) and lung cancer (71, 40%).

**Table 1:** Patient characteristics.

	number	%
Total	179	100
Male/Female		
Male	105	59
Female	74	41
Age (years)		
<50	29	16
50–75	130	73
>75	15	8
Unknown	5	3
Tumour type		
NSCLC	71	40
Melanoma	74	41
RCC	23	13
Urothelial	2	1
Other^a^	9	5

Table of patient demographic and tumour characteristics.

*NSCLC* non-small cell lung cancer, *RCC* renal cell carcinoma.

^a^Colorectal carcinoma, Merkel cell carcinoma, cervical carcinoma, gastric carcinoma, cutaneous squamous cell carcinoma.

Treatment details are summarised in Table 2. Fifty-eight (32%) had received prior RT and 101 (56%) had received prior SACT. Regarding SACT, 63 (35%) had received prior cytotoxic chemotherapy. A history of ipilimumab therapy would only have been applicable in the setting of the 74 melanoma patients, of whom 23 (31%) had a history of ipilimumab treatment. The median number of prior regimens was 1 (range 1 to 3).

**Table 2:** Treatment details.

	Number	%
Total	179	100
PD1 inhibitors		
Nivolumab	72	40
Pembrolizumab	89	50
Other^a^	18	10
Prior RT		
Yes	58	32
No	121	68
Prior cytotoxic		
Yes	63	35
No	116	65
Prior SACT		
Yes	101	56
No	78	44
Prior ipilimumab		
Yes	23	31
No	51	69

Treatment modality received and prior treatment history.

^a^Other: Atezolizumab, nivolumab/ipilimumab, durvalumab, cemiplimab, avelumab.

Regarding the specifics of the ICI treatment, 89 (50%) received pembrolizumab, 72 (40%) received nivolumab, 8 (5%) received atezolizumab, 7 (4%) received combined nivolumab/ipilimumab and one patient each (1%) received durvalumab, avelumab and cemiplimab. Median duration on therapy was 5.5 months (range 0.5 to 50.9).

Median follow-up time was 10.6 months with longest follow-up time of 56 months. One hundred patients (56%) had progression at some point, with median PFS 6.5 months (range 0.5 to 56.3 months). Eighty-four patients (47%) died during the study period.

**Fig. 1:**
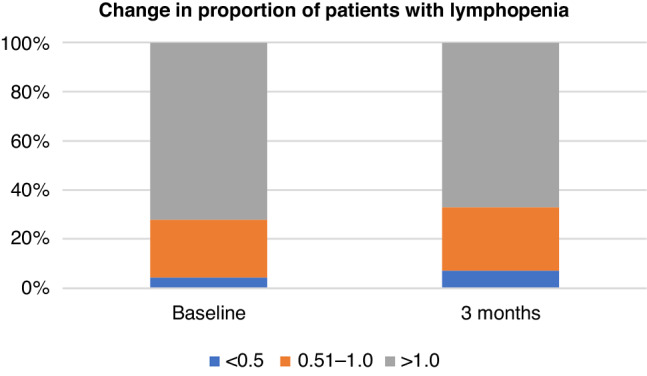
Change in proportion of patients with lymphopenia. Stacked barchart demonstrating percentage of cohort with normal ALCs, with decreased lymphocytes 0.51–1.0 × 10^9^cells/L and with lymphocytes less than 0.5 × 10^9^cells/L, at baseline and at 3 months from treatment initiation.

Regarding bloodwork, baseline and 3-month ALC are summarised in Fig. 1. 49 patients (27%) had a ALC of less than 1 × 10^9^cells/L at baseline (grade 1 and 2), and 8 (5%) had a ALC of less than 0.5 × 10^9^cells/L (grade 3 and 4). At 3 months, 56 (31%) had a ALC of less than 1 × 10^9^cells/L, and 13 (7%) had a ALC less than 0.5 × 10^9^ cells. Those with a history of RT had higher rates of lymphopenia: 30 of 58 (52%) were lymphopenic at baseline and 6 (10%) were severely lymphopenic. At 3 months, 28 (48%) were lymphopenic and 9 (16%) were severely lymphopenic.

**Fig. 2:**
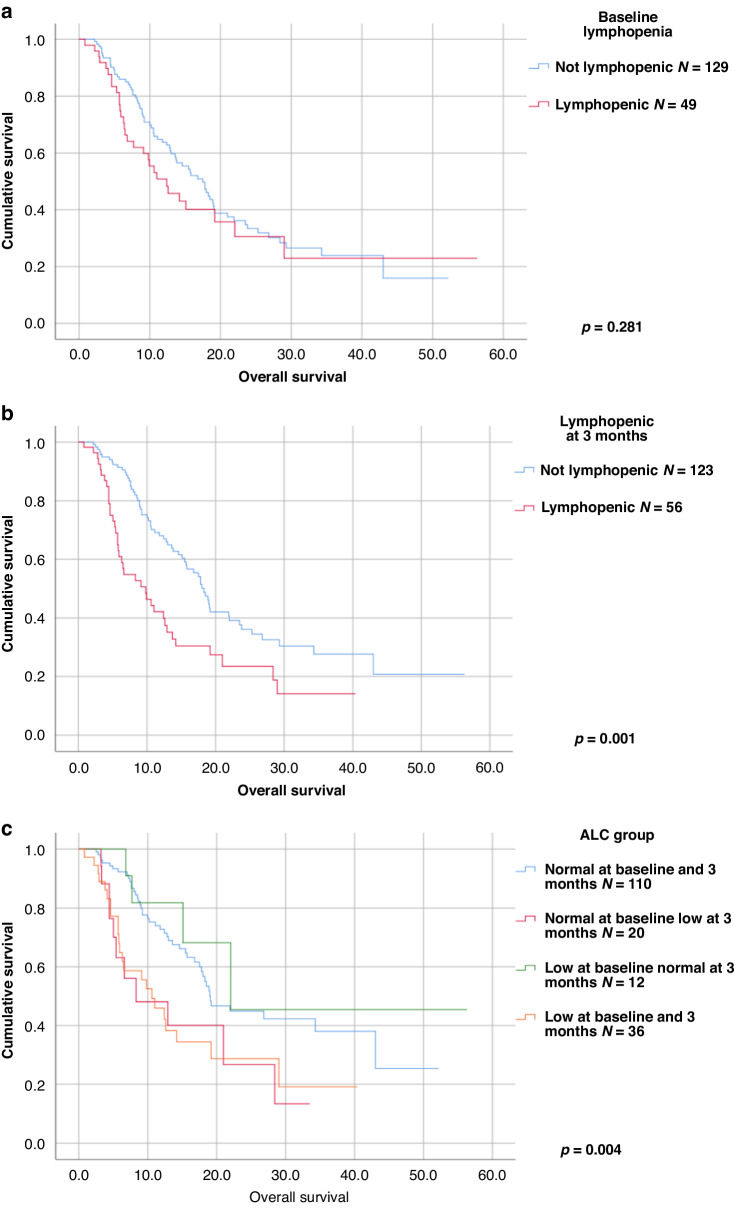
Kaplan–Meier survival plots for lymphocyte subsets. **a** Plot comparing patients with no baseline lymphopenia to those with baseline lymphopenia (<1 × 10^9^cells/L). **b** Plot comparing those who were lymphopenic at 3 months to those who were not. **c** Plot comparing patient groups NN, LN, NL, LL. ALC absolute lymphocyte count, NN normal ALC at baseline and at 3 months. LN low ALC at baseline, normal at 3 months. NL normal ALC at baseline, low at 3 months. LL low ALC at baseline and at 3 months.

With regard to the relationship between ALC and survival, outcomes are summarised in Table 3. Initially, outcomes were examined based on ALC at baseline and at 3 months. We found that those with baseline ALC of less than 1 × 10^9^cells/L had no significant difference in median PFS (6.5 vs 10.8 months, *p* = 0.482) or OS (12.4 vs 17.5 months, *p* = 0.281) (Fig. 2a) compared to those who were not lymphopenic. However, those who were lymphopenic at 3 months from treatment initiation had a significantly shorter PFS (5.3 vs 15.1 months, *p* = 0.005) and OS (9.8 vs 18.3 months, *p* = 0.001) than those who were not lymphopenic at 3 months (Fig. 2b). To account for the possible influence of immortal time bias on this outcome, a Cox regression analysis incorporating ALC as a time-dependent covariate was undertaken. This found that the difference in PFS did not reach statistical significance (*p* = 0.079) but the difference in OS was significant (*p* < 0.001).

**Table 3:** lymphocyte subsets.

Category	*n*	% of total	median PFS (mo)	*p* (log rank)	% without PD at 12 months	SE	low 95% CI	high 95% CI	median OS (mo)	*p* (log rank)	SE	low 95% CI		high 95% CI
ALC > 1000 at baseline	130	73	10.8	0.482	35	1.9	7.1	14.6	17.5	0.281	1.7	14.1	20.9	
ALC < 1000 at baseline	49	27	6.5		37	4.3	0	15	12.4		2.6	7.4	17.4	
ALC > 1000 at 3 months	123	69	15.1	0.005	39	3.3	8.6	21.6	18.3	0.001	1.1	16.2	20.4	
ALC < 1000 at 3 months	56	31	5.3		27	2	1.4	9.2	9.8		2.3	5.3	14.3	
baseline lymphopenia, persistent at 3 months	36	20	5.3	0.047	31	2.2	1.1	9.6	9.9		3.0	4.1	15.7	
no baseline or 3 month lymphopenia	107	60	12.3		37	3.5	5.5	19.2	18		1.5	15.1	20.9	
baseline lymphopenia, persistent at 3 months	36	20	5.3	0.081	31	2.2	1.1	9.6	9.9		3.0	4.1	15.7	
baseline lymphopenia with recovery at 3 months	12	7			50				22		6.5	9.2	34.8	
no lymphopenia at baseline or 3 months	107	60	13	0.024	38	3.1	6.9	19.1	18.3	0.01	1.05	16.2	20.4	
no lymphopenia at baseline with lymphopenia at 3 months	20	11	4.3		20	2.1	0.1	8.5	8.3		2.6	3.1	13.5	
baseline lymphopenia with recovery at 3 months	12	7	NR	0.48	50				22.0	0.548	6.6	9.1	34.9	
no baseline lymphopenia or at 3 months	107	60	13		38	3.1	6.9	19.1	18.3		1.1	16.2	20.4	
lymphopenia at baseline and at 3 months	36	20	5.3	0.708	31	2.2	1.1	9.6	9.9	0.72	3.0	4.1	15.7	
no baseline lymphopenia with lymphopenia at 3 months	20	11	4.3		20	2.1	0.1	8.5	8.3		2.6	3.1	13.5	

Table of estimates of median survival by Kaplan–Meier method and percentage without progression at 12 months for leukocyte subsets.

*ALC* absolute lymphocyte count, *PFS* progression-free survival, *OS* overall survival, *SE* standard error, *CI* confidence interval.

Subsequently, outcomes were analysed according to change in ALC over time. We grouped patients according to ALC at baseline and 3 months: NN (normal at baseline and three months), NL (normal at baseline, lymphopenic at 3 months), LN (lymphopenic at baseline, normal at 3 months), LL (lymphopenic at baseline and at 3 months). Figure 2c is a Kaplan–Meier curve comparing outcomes between these four groups.

Of note, when analysis was limited to LN and NN patients only, it was found there was no significant difference in PFS (NR vs 13.02 months, *p* = 0.48). In addition, NL patients had no significant difference in PFS from LL patients (4.3 vs 5.3 months, *p* = 0.708).

When analysis was limited to those with lymphopenia grade 3 and 4 (<0.5 × 10^9^cells/L), there were similar findings to all-grade lymphopenia. Those with severe lymphopenia at baseline had no significant difference in PFS compared to those without, but those with severe lymphopenia at 3 months had significantly shorter PFS than those without (3.6 vs 10.9 months, *p* = 0.026). This was further explored with a Cox regression analysis incorporating presence of severe lymphopenia as a time-dependent covariate. This found that the difference in PFS did not reach statistical significance (*p* = 0.214) but the difference in OS was significant (*p* = 0.006). This difference in OS remained significant on multivariable analysis incorporating age, sex, histologic subtype, previous RT, previous SACT, ICI type and whether the patient had an irAE.

On univariate analysis, a history of RT was significantly associated with baseline lymphopenia (*p* < 0.0005) but a history of SACT did not have such an association. Similarly, a history of RT was significantly associated with lymphopenia at 3 months (*p* = 0.001) but a history of SACT was not.

A logistic regression model that incorporated age, sex, histologic subtype, previous RT, previous SACT, prior ipilimumab, ICI type, and whether the patient had an irAE was used to identify significant predictors of lymphopenia. Lymphopenia at baseline was significantly associated with prior RT with an OR 7.78 (*p* < 0.0005) and with advancing age with an OR 1.065 (*p* = 0.004), indicating that for every year of advancing age, patients were 1.065 times more likely to be lymphopenic at baseline.

Regarding lymphopenia at 3 months, prior RT had a smaller effect that was still significant (OR 3.28, *p* = 0.002) as did advancing age (OR 1.038, *p* = 0.029). Sex, histologic subtype, prior SACT and the presence of irAE were not found to have a significant association with the presence of lymphopenia.

## Discussion

We describe the relationships between ALCs and survival outcomes for 179 patients treated with ICI in two hospitals.

This two-institution, retrospective study has found that, while baseline ALC does not appear prognostic for those on ICI, all-grade lymphopenia at 3 months is a significant negative prognostic marker. This appeared to be true both for those who had been lymphopenic at baseline, and those who had previously had normal ALCs. Although both groups had inferior outcomes to patients with normal 3-month ALCs, they did not differ significantly from one another, reinforcing that the baseline count is of less prognostic importance

Conversely, while those who were not lymphopenic at any point may reasonably have been predicted to have had the best outcomes, they in fact did not differ significantly from patients with baseline lymphopenia who recovered counts at 3 months. These findings were consistently true when the analysis was limited to those with severe lymphopenia (grade three and four).

Consistent with previous work in the area, it was found that one of the strongest predictors of future lymphopenia was a history of RT, whereas a history of SACT did not have a significant impact [23]. The relationship with a history of RT was found with ALC both at baseline and 3 months, and when controlling for other factors. Advancing age also had a significant association with lymphopenia at both points, although with a smaller effect size.

The mechanism by which lymphopenia could cause poorer survival for patients on ICI has not been conclusively established. Possible explanations include the central role of lymphocytes in the immunological synapse; that the low ALC could be associated with a pre-existing immunosuppressed condition, leading to an inadequate immunological reaction [13]; lymphopenia could represent inflammation or other factors associated with advanced disease, and therefore be a surrogate marker of poor prognosis [15]; or that lymphopenia could represent immune exhaustion, with attenuated anti-tumour effect [24].

The value of ALC as a prognostic marker after initiation of ICI has previously been investigated. In the setting of anti-CTLA-4 antibodies, both decline in ALC after initiation of ipilimumab (any negative ALC slope) [25] and drop below an absolute threshold (<1 × 10^9^cells/L) [26] have been associated with inferior survival. Low ALC after treatment initiation has also been associated with inferior survival in the setting of anti-PD(L)1 antibodies [15, 27]. It is not clear why ALC after treatment initiation would perform better as a prognostic marker than ALC at baseline. This may be because baseline low ALC reflects more transient toxicity from recent treatment (such as RT) whereas ALC at an interval after treatment initiation reflects more advanced disease or more persistent toxicity.

Grossman et al. have previously found that, 2 months after initiating chemoradiation, 43% of patients had severe and persistent lymphopenia (<0.5 × 10^9^cells/L) [12]. While our rates of severe lymphopenia at baseline were lower than this, our study population was different in that it was not uniquely composed of patients with a history of combined chemoradiation. Lymphopenia following chemoradiation has also been identified as a poor prognostic marker for those who subsequently go on to consolidative immunotherapy, conferring a worse PFS than in non-lymphopenic patients [28].

Various characteristics of RT have been associated with poorer outcomes. Multiple courses of RT, multiple irradiated sites and high RT doses (≥50 Gy in 2 Gy equivalent doses) have previously been found to increase risk of lymphopenia in a multivariate analysis [29]. A further study looking specifically at the impact of palliative RT in cancer patients starting immunotherapy found extracranial or prolonged courses of RT to be associated with severe lymphopenia (OR 3.7, *p* = 0.001 and OR 3.9, *p* = 0.001), and subsequently with poorer survival on ICI (HR 2.1, *p* = 0.03) [30].

This study has several limitations. It is a retrospective study, which was dependent on completeness of patient records and cannot control for patient selection. Data is from two institutions only, and therefore subject to the risk of local influences. It may also be subject to time bias: patient data is included from as early as 2014, when immunotherapy was less frequently used in the first line setting, which may have had an impact on outcomes recorded. However, the choice of time period during which data was collected also has some strengths: as patients were mostly treated with single-agent ICI regimens at that time rather than combination chemotherapy-ICI approaches, the results are not distorted by the effect of concurrent chemotherapy.

The dose and site of radiotherapy was not recorded, but radiation was predominantly palliative in intent.

## Conclusion

All-grade lymphopenia at 3 months from treatment initiation, whether new or persisting, is a significant negative prognostic marker for cancer patients on immunotherapy. Lymphopenia at baseline does not have the same prognostic implications. A history of RT is a significant risk factor for lymphopenia at baseline and at three months from treatment initiation. Prospective validation of these results is required among cancer patients on immunotherapy.

Comparison of ALC with NLR, PLR and other haematologic indices in this and other cohorts would be of benefit to establish the optimal prognostic biomarker.

In addition, it is unknown whether ALC during adjuvant immunotherapy is also prognostic of outcome, and this merits further investigation either prospectively or retrospectively. Clinicians should be vigilant for early signs of progression in those with a history of RT, and those with persisting or new lymphopenia at 3 months from treatment initiation.

## Data Availability

The datasets generated and analysed during the study are available from the corresponding author on request.
